# SIRT1 Suppresses the Senescence-Associated Secretory Phenotype through Epigenetic Gene Regulation

**DOI:** 10.1371/journal.pone.0116480

**Published:** 2015-01-30

**Authors:** Tomohisa Hayakawa, Mika Iwai, Satoshi Aoki, Koichi Takimoto, Mitsuo Maruyama, Wakako Maruyama, Noboru Motoyama

**Affiliations:** 1 Department of Cognitive Brain Science, Research Institute, National Center for Geriatrics and Gerontology, Obu, Aichi, Japan; 2 Department of Aging Research, Nagoya University Graduate School of Medicine, Nagoya, Aichi, Japan; 3 Department of Bioengineering, Nagaoka University of Technology, Nagaoka, Niigata, Japan; 4 Department of Mechanism of Aging, Research Institute, National Center for Geriatrics and Gerontology, Obu, Aichi, Japan; University of Texas Health Science Center at San Antonio, UNITED STATES

## Abstract

Senescent cells develop a pro-inflammatory response termed the senescence-associated secretory phenotype (SASP). As many SASP components affect surrounding cells and alter their microenvironment, SASP may be a key phenomenon in linking cellular senesence with individual aging and age-related diseases. We herein demonstrated that the expression of Sirtuin1 (SIRT1) was decreased and the expression of SASP components was reciprocally increased during cellular senescence. The mRNAs and proteins of SASP components, such as IL-6 and IL-8, quickly accumulated in SIRT1-depleted cells, and the levels of these factors were also higher than those in control cells, indicating that SIRT1 negatively regulated the expression of SASP factors at the transcriptional level. SIRT1 bound to the promoter regions of IL-8 and IL-6, but dissociated from them during cellular senescence. The acetylation of Histone H3 (K9) and H4 (K16) of the IL-8 and IL-6 promoter regions gradually increased during cellular senescence. In SIRT1-depleted cells, the acetylation levels of these regions were already higher than those in control cells in the pre-senescent stage. Moreover, these acetylation levels in SIRT1-depleted cells were significantly higher than those in control cells during cellular senescence. These results suggest that SIRT1 repressed the expression of SASP factors through the deacetylation of histones in their promoter regions.

## Introduction

Cellular senescence is a state of irreversible cell cycle arrest that is caused by various cellular stresses, such as telomere shortening, oxidative stress, DNA damage, and the aberrant activation of oncogenes [1,2]. Senescent cells have been shown to accumulate in several diverse precancerous tissues from both humans and mice and are progressively lost during cancer progression [3–7], thereby revealing senescence as a potent initial barrier to cancer progression. Senescence also prevents fibrosis and other diseases caused by hyperproliferation in a cell autonomous manner [8,9]. In addition to this cell autonomous function, senescent cells secrete a number of soluble factors associated with inflammation, growth, and modulation of the extracellular matrix as a cell non-autonomous function, a phenotype termed the senescence associated secretory phenotype (SASP) or senescence messaging secretome (SMS) [2,10]. IL-6 and IL-8, key components of SASP, reinforce senescence in neighboring cells, committing them to either malignant transformation or senescence in order to facilitate the suppression of tumors [11,12]. Moreover, some SASP components, such as IL-1β, can induce senescence in normal neighboring cells in a paracrine manner [13,14]. However, some SASP components are also known to stimulate phenotypes associated with aggressive cancer cells [15]. Furthermore, some SASP components, including matrix metalloprotease, may modulate tissue architecture by cleaving extracellular matrix proteins or other components in the tissue microenvironment [16]. Thus, SASP affects neighboring and surrounding cells in autocrine and paracrine manners, thereby altering their microenvironment. As senescent cells accumulate with age, SASP may be a key phenomenon linking cellular senescence with functional decline in tissues and organs, individual aging, and age-associated diseases.

The persistent DNA damage response (DDR) is crucial for the induction and maintenance of SASP [10]. Many SASP components are regulated by the transcription factors, nuclear factor κB (NF-κB) and CCAAT/enhancer-binding protein (C/EBP) β, at the transcriptional level [2,11,17,18]. Moreover, the DDR-mediated proteasome-dependent degradation of the methyltransferases, G9a and GLP, through the activation of APC/C-Cdh1 promotes the expression of SASP components, indicating that SASP is also regulated by DDR-mediated epigenetic gene regulation [19]. However, the precise regulatory mechanism responsible for SASP currently remains unknown.

Sirtuin1 (SIRT1) is an NAD^+^-dependent protein deacetylase, which regulates a diverse set of biological processes by deacetylating transcription factors, histones, repair enzymes, and other cellular proteins. SIRT1 plays a pivotal role in protection against various age-related diseases such as neurodegenerative diseases, cardiovascular diseases, cancer, and metabolic syndromes in mammals [20,21]. Although previous studies reported that overexpression of the SIRT1 ortholog in the yeast, *Caenorhabditis elegans*, *Drosophila melanogaster* and mouse extended their lifespans, its effects on longevity *per se* remain controversial [22–26]. SIRT1 regulates gene expression by deacetylating transcription factors, including p53, Forkhead box-O (FOXO), and NF-κB, thereby modifying their activity directly [27–31]. SIRT1 has also been shown to repress gene expression by silencing the chromatin structure through its histone deacetylation activity [32]. SIRT1 represses repetitive DNA and several sets of genes that cross the whole genome by binding to and deacetylating histones. During aging or in response to DNA damage, SIRT1 relocalizes to DNA damage sites, thereby derepressing the genes of SIRT1-mediated silencing loci [32]. This DNA damage-induced redistribution of SIRT1 results in changes to the transcriptome through epigenetic modifications that parallel those in the mouse brain during aging.

In the present study, we showed that the depletion of SIRT1, but not its overexpression, accelerated and enhanced the expression of SASP components at the transcriptional level. SIRT1 bound to the promoter regions of SASP components, but dissociated from these regions in response to DNA damage, a key senescence stimulus. The depletion of SIRT1, but not its overexpression, enhanced the acetylation levels of histone H3 and H4 on the promoter regions of SASP components even in cells at the pre-senescence stage and those undergoing senescence. These results demonstrated that SIRT1 regulated the expression of SASP components through epigenetic gene regulation.

## Materials and Methods

### Cell culture

The primary human embryonic lung fibroblast, MRC-5, was purchased from ATCC. MRC-5/TERT were immortalized by introducing the human telomerase reverse transcriptase (hTERT). The human skin fibroblast BJ/TERT cell lines were kindly provided by Hahn WC [33]. Cells were cultured in Dulbecco’s modified Eagle’s medium (DMEM) supplemented with 10% fetal bovine serum, 1 mM sodium pyruvate, 0.1 mM nonessential amino acids, 2 mM L-glutamine, penicillin (100 U/ml), streptomycin (100 μg/ml), and 50 μM 2-mercaptoethanol.

### Transfection and infection

The siRNA targeting human SIRT1 (SIRT1VHS50608 [VALIDATED], 50609 [VALIDATED]) and non-targeting siRNA pool siControl (siGENOME siRNA pool #1, D-001206–13) were purchased from Invitrogen and ThermoScientific.

Cells were transfected with siRNA using Lipofectamine RNAi/MAX according to the manufacturer’s instructions (Invitrogen). The culture medium was changed to normal medium 2 hours after transfection.

The retrovirus vectors containing the shRNA sequence against SIRT1 (pSuper-Retro-Puro-shSIRT1-HS11) or hSIRT1 (pBabe-INeo-hSIRT1) [34], and Plat-A retroviral packaging cells [35] were kindly provided by Vaziri H and Kitamura T, respectively. To produce retroviruses, we transfected Plat-A cells with pSuper or pBabe using FuGENE 6 (Promega). The resulting retroviruses were used to infect fibroblasts, were then selected in medium containing puromycin (2μg/ml) or G418 (1mg/ml), which results in SIRT1-knockdown (MRC5-shSIRT1, MRC-5/TERT-shSIRT1, BJ/TERT-shSIRT1) or SIRT1-overexpressing cells (MRC-5-SIRT1, MRC-5/TERT-SIRT1, BJ/TERT-SIRT1) being obtained.

### Immunoblot analysis

Cells were lysed in a solution containing 50 mM Tris-HCl (pH 7.5), 125 mM NaCl, 1.0% Nonidet P-40 (Nacalai Tesque), a mixture of protease inhibitors (Complete mini, Roche), and a mixture of phosphatase inhibitors (PhosSTOP, Roche). The protein concentration of the lysate was determined with the BCA protein assay reagent (Pierce), after which samples were subjected to SDS–polyacrylamide gel electrophoresis and immunoblot analysis with mouse monoclonal antibodies to human SIRT1 (1:1000; Cell Signaling Technology), mouse polyclonal antibodies to human IL-8 (1:1000; R&D), rabbit polyclonal antibodies to human IL-6 (1:1000; Abcam), mouse polyclonal antibodies to human γ-tubulin (1:1000; Sigma Aldrich). Immunocomplexes were visualized with horseradish peroxidase-conjugated secondary antibodies (BIO-RAD) and the ECL prime system (GE Healthcare), and were then detected using ImageQuant LAS 4000 (GE Healthcare).

### Quantitative RT-PCR analysis

Total RNA was extracted using TRIzol reagent (Invitrogen) according to the manufacturer’s instructions, and portions of RNA (1 μg) were subjected to reverse transcription (RT) with the QuantiTect Reverse Transcription Kit (QIAGEN). The cDNA synthesized was subjected to real-time polymerase chain reaction (PCR) analysis using the THUNDERBIRD SYBR qPCR Mix (TOYOBO) in the CFX96 real-time PCR system (BIO-RAD). PCR was performed using the following primers.

IL-8; Forward: 5′-TTGGCAGCCTTCCTGATTTC-3′ and Reverse: 5′-TCTTTAGCACTCCTTGGCAAAAC-3′, IL-6; Forward: 5′-CCAGGAGCCCAGCTATGAAC-3′ and Reverse: 5′-CCCAGGGAGAAGGCAACTG-3′, IL-1β; Forward: 5′-GGCCCTAAACAGATGAAGTGCT-3′ and Reverse: 5′-TGCCGCCATCCAGAGG-3′, Growth-related oncogene (GRO)-α: Forward: 5′-TTCACCCCAAGAACATCCAA-3′ and Reverse: 5′-CTCCTAAGCATGCTCAAACAC-3′, GAPDH; Forward: 5′-GAAGGTGAAGGTCGGAGTC-3′ and Reverse: 5′-GAAGATGGTGATGGGATTTC-3′

### Chromatin immunoprecipitation (ChIP) assay

ChIP analysis was performed using the simpleChIP Enzymatic Chromatin IP Kit (Cell Signaling Technology). Briefly, cells were crosslinked with 1% formaldehyde and nuclear DNA was then digested with micrococcal nuclease. The chromatin fractions were extracted. The resulting samples were subjected to immunoprecipitation using polyclonal antibodies to Acetyl-histone H3 (5μl/assay, millipore), Acetyl-histone H4 (5μl/assay; millipore), or SIRT1 (5μl/assay, Cell Signaling Technology). The immunoprecipitated DNAs were quantified by real-time RT-PCR analysis. PCR was performed using the following primers.

IL-8 promoter region; Forward: 5′-GGTTTGCCCTGAGGGGATG-3′ and Reverse: 5′-ACAGAGCTGCAGAAATCAGGAAGGCT-3′, IL-6 promoter region; Forward: 5′-AATGTGGGATTTTCCCATGA-3′ and Reverse: 5′-GCTCCTGGAGGGGAGATAGA-3′.

## Results

### The reduction of SIRT1 preceded the expression of SASP components

**Figure 1:**
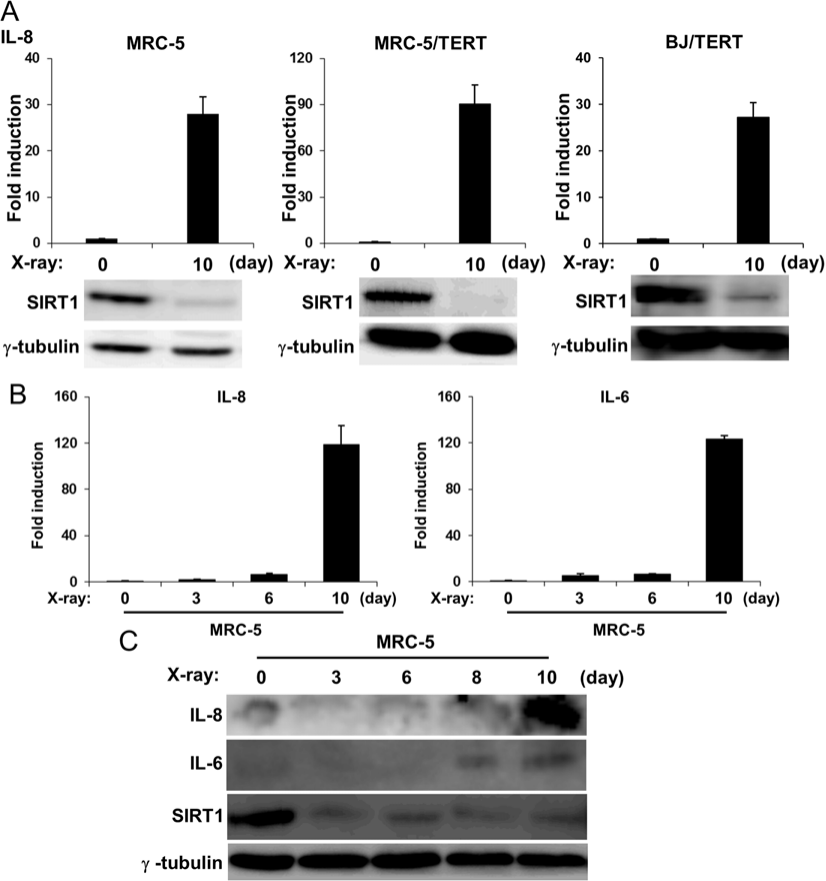
The expression of SASP components and SIRT1 during cellular senescence. **A**, The indicated cells were exposed (or not) to 10 Gy of X-radiation, cultured for 10 days, and then total RNA and cell lysates were extracted. IL-8 and IL-6 mRNA levels were determined by quantitative RT-PCR analysis with normalization to GAPHD mRNA levels and expressed as fold induction by x-radiation. Data are means ± s.d. of triplicates from experiments that were repeated at least twice with similar results. Cell lysates were subjected to immunoblot analysis using antibodies to SIRT1 and **γ**-tubulin. **B and C**, MRC-5 human fibroblast cells were treated as in **A**, Total RNA and cell lysates were extracted at indicated days. IL-8 and IL-6 mRNA levels were then determined by quantitative RT-PCR analysis (B). The levels of the indicated mRNA were normalized to that of GAPDH mRNA and shown as fold induction by 0h. Data are means ± s.d. of triplicates from experiments that were repeated at least twice with similar results. Cell lysates were subjected to immunoblot analysis using antibodies to IL-8, IL-6, SIRT1, and **γ**-tubulin. **γ**-Tubulin was used as a loading control (C).

We used human fibroblasts, cells that had been characterized in detail, in the senescence study in order to observe SASP. IL-8 mRNA, one of the SASP components, was highly induced in senescent MRC-5 cells that had exposed to x-radiation.Consistent with previous reports [12,36–39], the hTERT-mediated immortalized MRC-5 (MRC-5/TERT) and BJ (BJ/TERT) cell lines underwent senescence and the induction of IL-8 mRNA was also observed upon x-radiation (Fig. 1A). To elucidate the regulatory mechanism of SASP during cellular senescence, we first monitored the expression of several proteins involved in regulating the aging process or age-related diseases. We found that the expression of the SIRT1 protein was reciprocally decreased in senescent MRC-5 cells (Fig. 1A). The down-regulation of the SIRT1 protein was also observed in senescent MRC-5/TERT and BJ/TERT cells, indicating that these reductions did not depend on the expression of hTERT (Fig. 1A). The expression of IL-8 and IL-6 mRNAs and proteins was increased after the induction of senescence (Fig. 1B and C). In contrast, expression of the SIRT1 protein was decreased 3 days after the induction of senescence (Fig. 1B). These results indicated that the reduction in the SIRT1 protein preceded the induction of SASP components, regardless of the expression of hTERT.

### The depletion of SIRT1 accelerated and enhanced SASP

**Figure 2:**
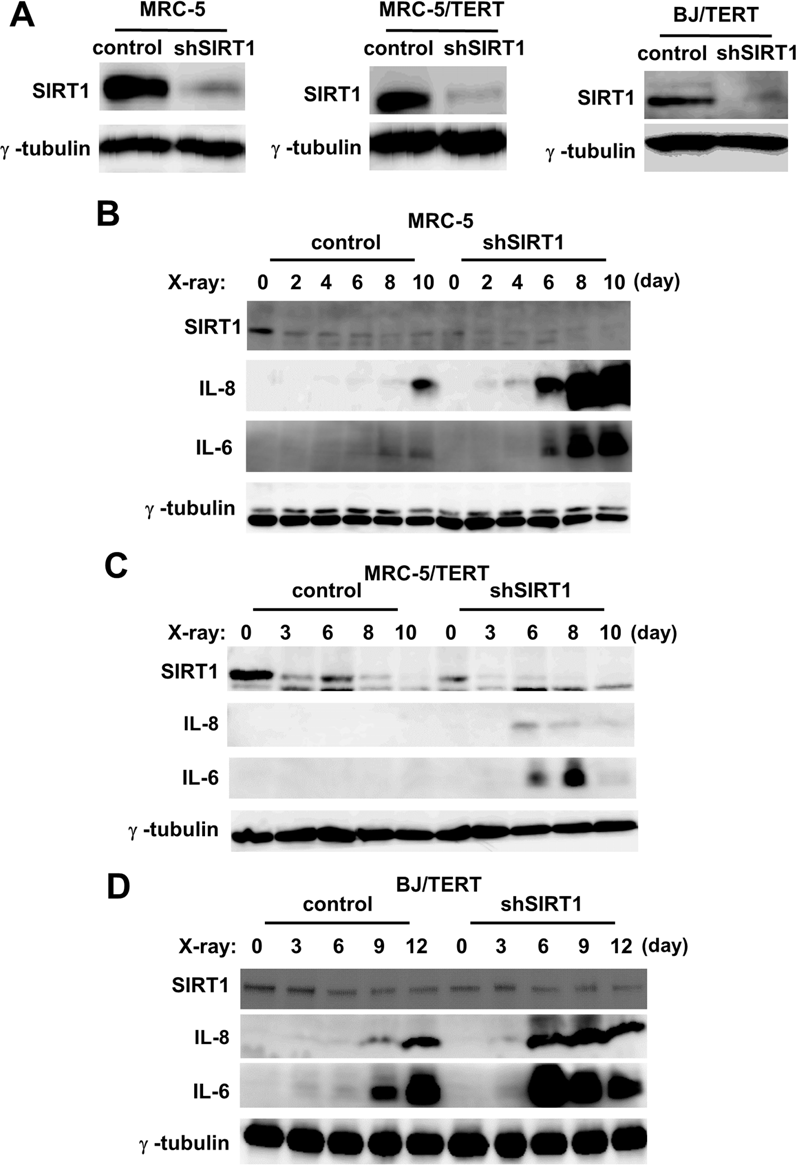
Effects of stable SIRT1 knockdown on protein expression of SASP components. **A**, MRC-5, MRC-5/TERT, and BJ/TERT cells were infected with a control retrovirus or the retrovirus expressing shSIRT1 and then selected for 4 days. The resulting cells were lysed and then subjected to immunoblot analysis with antibodies to SIRT1 and **γ-**tubulin (loading control). **B**, **C**, and **D**, SIRT1 knockdown and control cells **(B**: MRC-5 **C**: MRC-5/TERT **D**: BJ/TERT) were exposed (or not) to 10Gy of X-irradiation. Cell were lysed on the indicated days after irradiation, and then subjected to immunoblot analysis using antibodies to IL-8, IL-6, SIRT1, and **γ**-tubulin. **γ**-Tubulin was used as a loading control.

The reciprocal expression of SIRT1 and SASP components revealed that SIRT1 regulated SASP. To examine this in more detail, we first investigated the expression of SASP components during cellular senescence in SIRT1 knockdown cells. We established SIRT1 depleted and control cells that stably express shRNA as a result of infection with a recombinant retrovirus. The depletion of SIRT1 in MRC-5, MRC-5/TERT, and BJ/TERT cells was confirmed by western blot analysis (Fig. 2A). The expression of the SIRT1 protein was decreased in control MRC-5 cells from 2 days and sustained during senescence. No significant differences were observed in the expression of the SIRT1 protein between SIRT1-depleted MRC-5 cells and senescent control cells (at 10 days); however, further reductions were noted during senescence (Fig. 2B). We then monitored the expression of the SASP components, the IL-8 and IL-6 proteins. Although the expression of the IL-8 and IL-6 proteins was increased, starting from day 8 in control MRC-5 cells, the induction of these proteins was already observed 4 or 6 days after the induction of senescence in SIRT1-depleted MRC-5 cells. Furthermore, the expression of the IL-8 and IL-6 proteins was markedly increased in SIRT1-depleted MRC-5 cells (Fig. 2B). Similar results were noted in SIRT1-depleted MRC-5/TERT and BJ/TERT cells (Fig. 2C and D). Thus, these results suggested that the depletion of SIRT1 accelerated and enhanced the induction of SASP components.

### The depletion of SIRT1 accelerated and enhanced the expression of SASP components at the transcriptional level

**Figure 3:**
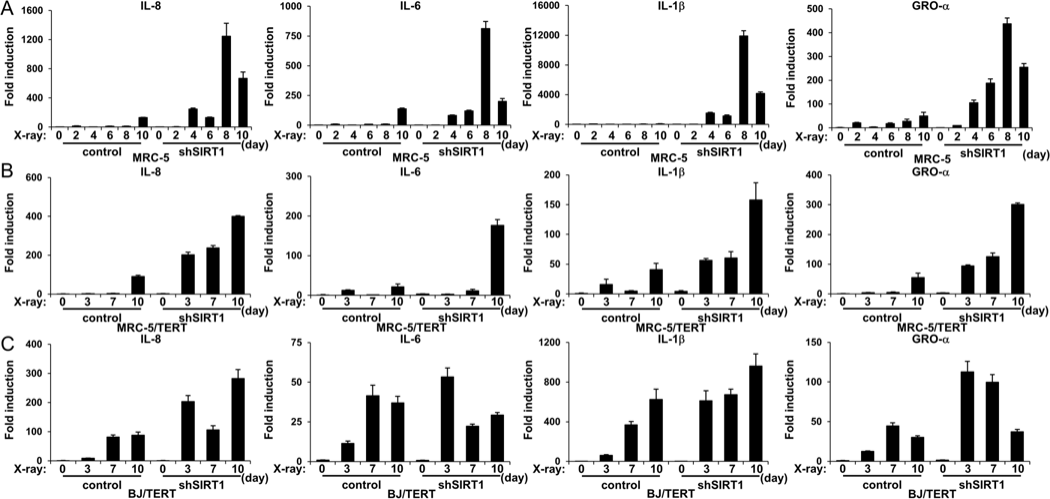
Effects of stable SIRT1 knockdown on mRNA expression of SASP components. **A**, **B**, and **C**, The indicated cells were exposed (or not) to 10Gy of X-irradiation. Total RNA was extracted on the indicated days after irradiation. I IL-8, IL-6, IL-1β, and GRO-α mRNA levels were then determined by quantitative RT-PCR analysis. The levels of the indicated mRNA were normalized to that of GAPDH mRNA and shown as fold induction by 0h. Data are means ± s.d. of triplicates from experiments that were repeated at least twice with similar results.

A previous study reported that the protein expression of SASP components was not always directly related to its mRNA expression [16]. Therefore, we examined the mRNA levels of the SASP components, IL-8, IL-6, IL-1β and GRO-α, in these cells using real time quantitative-PCR. Consistent with the results for protein expression, although the mRNA levels of IL-8, IL-6, IL-1β, and GRO-α increased starting from 6 days in control MRC-5 cells, the induction of these mRNAs could already be detected by 4 days after the induction of senescence in SIRT1-depleted MRC-5 cells. Furthermore, the levels of these mRNAs were markedly increased in SIRT1-depleted MRC-5 cells (Fig. 3A). These phenomena were also observed in SIRT1-depleted MRC-5/TERT and BJ/TERT cells (Fig. 3B and C). Taken together, these results indicated that the depletion of SIRT1 accelerated and enhanced the expression of SASP components at the transcription level.

### Transient knockdown of SIRT1 was sufficient to promote SASP

**Figure 4:**
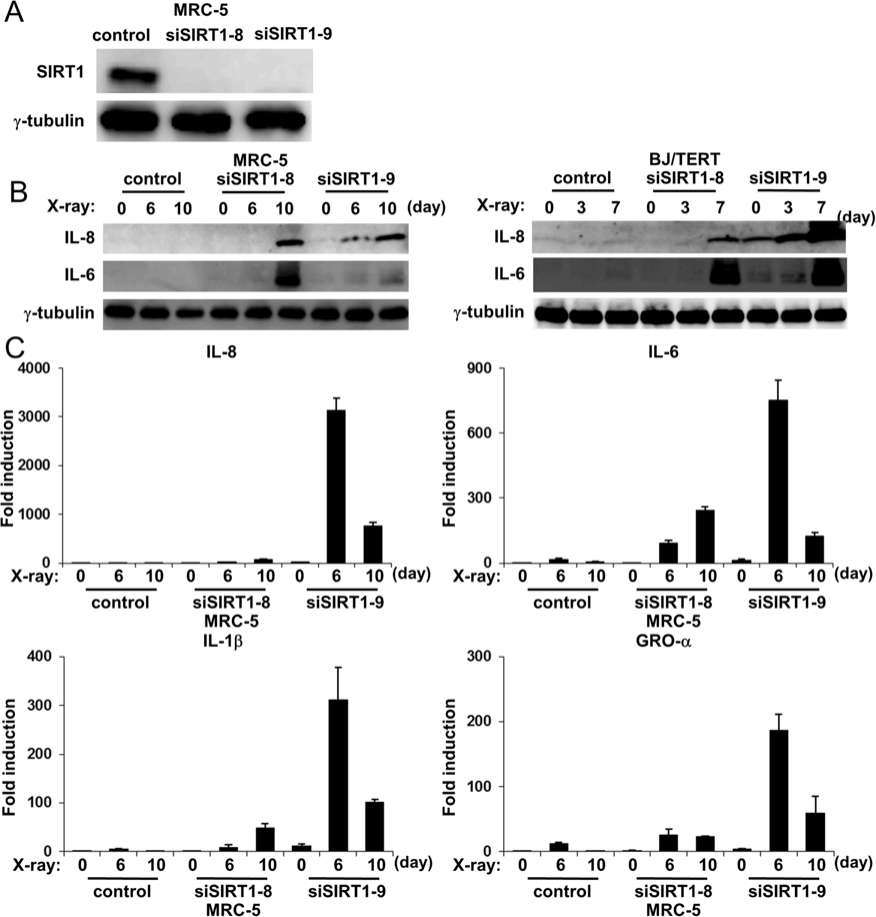
Effects of transient SIRT1 knockdown on expression of SASP components. **A**, MRC-5 cells were transfected with siRNA against SIRT1 (siSIRT1–8 and siSIRT1–9) or control (siControl), and then lysed 2 days after transfection. Cell lysates were subjected to immunoblot analysis using antibodies to SIRT1 and **γ**-tubulin (loading control). **B**, MRC-5 cells were transfected as in A and cultured for 2 days. The resulting cells were exposed (or not) to 10Gy of X-radiation. Cells were lysed on the indicated days after irradiation and subjected to immunoblot analysis using antibodies to IL-8, IL-6, and γ-tubulin. **γ**-Tubulin was used as a loading control. **C**, SIRT1 knockdown and control cells were exposed (or not) to 10Gy of X-irradiation. Total RNA was extracted on the indicated days after irradiation. IL-8, IL-6, IL-1β, and GRO-α mRNA levels were then determined by quantitative RT-PCR analysis. The levels of the indicated mRNA were normalized to that of GAPDH mRNA and shown as fold induction by 0h. Data are means ± s.d. of triplicates from experiments that were repeated at least twice with similar results.

In the above experiments using stably SIRT1-depleted cells, marked differences were observed in SIRT1 protein expression levels between control and SIRT1-depleted cells in untreated, pre-senescent stage cells, suggesting that SIRT1 may play a role at the pre-senescent stage. To examine this possibility, we introduced siRNA against SIRT1 (siSIRT-8 and siSIRT1–9) and the non-targeting siRNA pool (siControl) into MRC-5 cells. Both sets of siSIRT1 significantly depleted the expression of SIRT1 (Fig. 4A). In accordance with stable SIRT1-depleted MRC-5 cells, IL-8 and IL-6 protein levels were markedly increased in temporal SIRT1-knockdown MRC-5 cells. Furthermore, IL-8, IL-6, IL-1β and GRO-α mRNA levels were also markedly increased with accelerated kinetics in temporal SIRT1-knockdown MRC-5 cells (Fig. 4B). These results revealed that a deficit in SIRT1 at untreated, pre-senescent stage cells, i.e., when cells were subjected to senescence stimuli, was sufficient to promote SASP.

### Ectopic expression of SIRT1 did not suppress SASP

**Figure 5:**
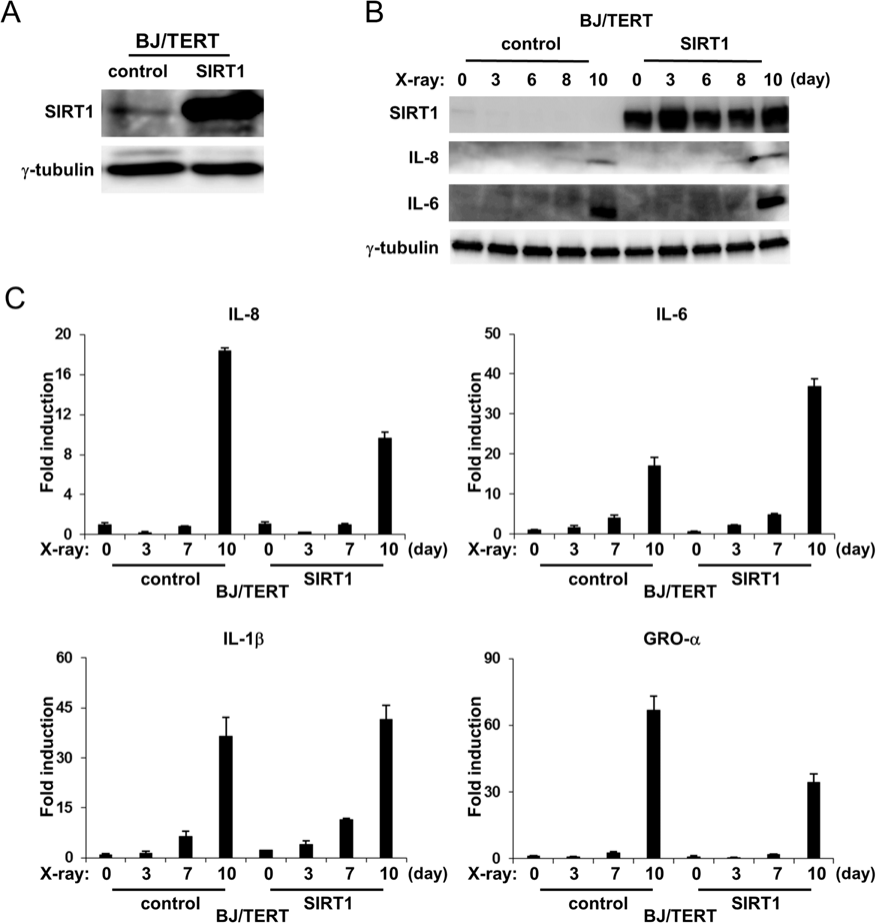
Effects of SIRT1 overexpression on expression of SASP components. **A**, BJ/TERT cells were infected with a control retrovirus or the retrovirus expressing SIRT1 and then selected for 4 days. The resulting cells were lysed and then subjected to immunoblot analysis with antibodies to SIRT1 and **γ**-tubulin (loading control). **B**, SIRT1-overexpressing and control cells were exposed (or not) to 10Gy of X-irradiation. Cell were lysed on the indicated days after irradiation, and then subjected to immunoblot analysis using antibodies to IL-8, IL-6, SIRT1, and **γ**-tubulin. **γ**-Tubulin was used as a loading control. **C**, SIRT1-overexpressing and control cells were exposed (or not) to 10Gy of X-irradiation. Total RNA was extracted on the indicated days after irradiation. IL-8, IL-6, IL-1β, and GRO-α mRNA levels were then determined by quantitative RT-PCR analysis. The levels of the indicated mRNA were normalized to that of GAPDH mRNA and shown as fold induction by 0h. Data are means ± s.d. of triplicates from experiments that were repeated at least twice with similar results.

We determined whether the ectopic expression of SIRT1 could suppress SASP. We established BJ/TERT cells that stably expressed SIRT1 as a result of infection with a recombinant retrovirus. A significantly higher level of the SIRT1 protein was observed in SIRT1-expressing BJ/TERT cells than in control cells (Fig. 5A). Although SIRT1 protein levels were decreased in control cells, the expression of SIRT1 was sustained at a higher level during the induction of senescence in SIRT1-expressing BJ/TERT cells (Fig. 5B). IL-8 and IL-6 protein levels in SIRT1-expressing BJ/TERT cells were similar to those in control cells during the induction of senescence (Fig. 5B). Consistent with the results for protein expression, IL-8, IL-6, IL-1β, and GRO-α mRNA levels were also equal to those in control cells (Fig. 5C). These results showed that the ectopic expression of SIRT1 was not sufficient to suppress SASP in spite of the sustained expression of ectopic SIRT1 during the induction of senescence.

### SIRT1 repressed SASP components in an epigenetic manner

**Figure 6:**
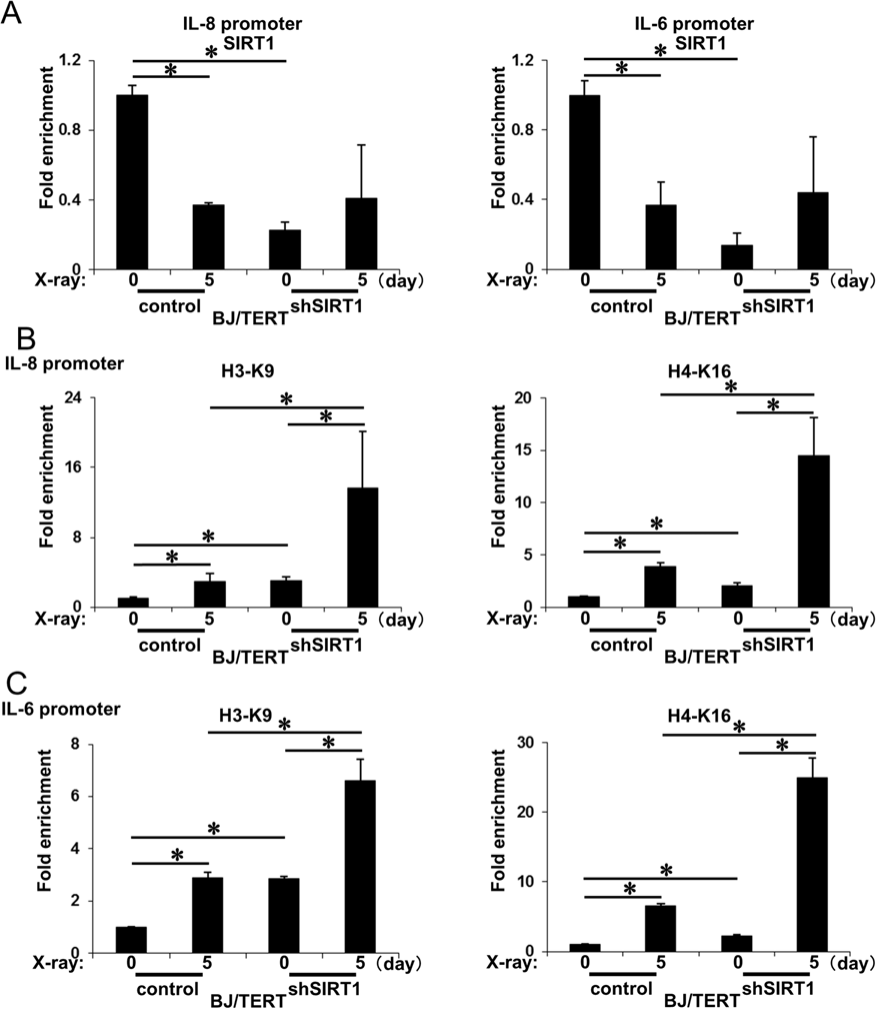
SIRT1 binding and histone acetylation on promoter regions of SASP component genes in SIRT1-depleted cells. **A**, SIRT1 knockdown and control BJ/TERT cells were subjected (or not) to 10 Gy of X-radiation. On the indicated days after irradiation, cells were processed for ChIP analysis using antibodies to SIRT1. The levels of the promoter regions of IL-8 (left) and IL-6 (right) were determined by quantitative RT-PCR analysis and shown as fold enrichment by 0h. Data are means ± s.d. of triplicates from experiments that were repeated at least twice with similar results. ∗, p<0.05 Student’s *t*-test. **B and C**, Cells were treated as in A and processed for ChIP analysis using antibodies to H3-K9ac (left) or H4-K16ac (right). The levels of the promoter regions of IL-8 (B) and IL-6 (C) were determined by quantitative RT-PCR analysis and shown as fold enrichment by 0h. Data are means ± s.d. of triplicates from experiments that were repeated at least twice with similar results. ∗, p<0.05 Student’s t-test.

SIRT1 was previously shown to repress several genes by regulating the chromatin structure through its histone deacetylase activity [32]. Although SIRT1 is known to bind to promoter regions to repress gene expression by SIRT1-repressing genes, it has also been shown to relocalize into DNA damage sites in response to DNA damage, thereby derepressing and inducing the expression of SIRT1-repressing genes [32]. These findings suggested that SIRT1 repressed the expression of SASP components by epigenetically modifying their promoter regions. To examine this possibility in more detail, we monitored the binding of SIRT1 to and histone acetylation status of the promoter regions of IL-8 and IL-6 genes by chromatin immunoprecipitation. Although SIRT1 bound to the promoter regions of the IL-8 and IL-6 genes, SIRT1 dissociated from those regions upon the induction of senescence in control cells (Fig. 6A). In accordance with SIRT1 binding, the acetylation levels of histone H3 (K9) and H4 (K16) on the promoter regions of IL-8 and IL-6 genes were markedly increased during the induction of senescence (Fig. 6B). The binding of SIRT1 to these regions was markedly lower in SIRT1-depleted cells than in control cells. However, the amount of SIRT1 on these regions in SIRT1-depleted cells was similar to that in control cells when senescence was induced (Fig. 6A). Even at the untreated, pre-senescence stage, the acetylation levels of histone H3 (K9) and H4 (K16) on the promoter regions of IL-8 and IL-6 genes in SIRT1-depleted cells were already significantly higher than those in control cells. These acetylation levels were also higher than those in control cells with the induction of senescence (Fig. 6B). These results showed that SIRT1 bound to and repressed the promoter regions of SASP components, and also that SIRT1 dissociated from these regions with the induction of senescence, thereby inducing the expression of SASP components.

### Ectopic SIRT1 dissociated from promoter regions during the induction of senescence

**Figure 7:**
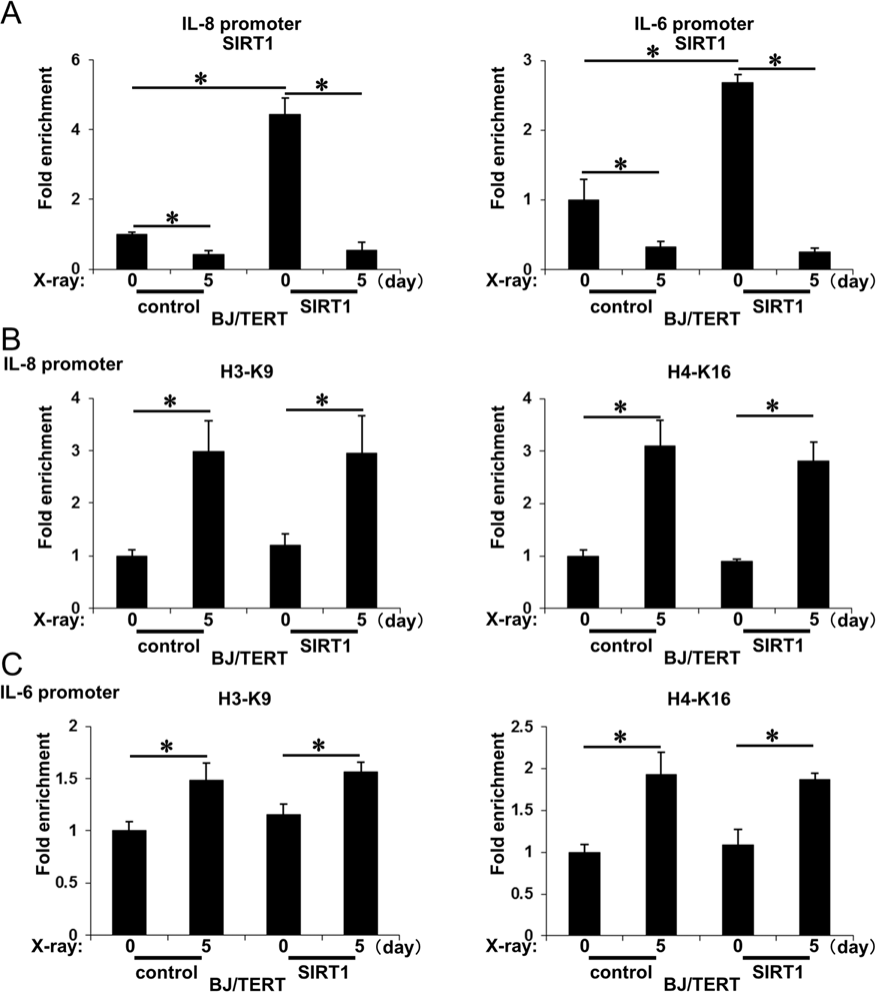
SIRT1 binding and histone acetylation on promoter regions of SASP component genes in SIRT1-overexpressing cells. **A**, SIRT1-overexpressing and control BJ/TERT cells were subjected (or not) to 10 Gy of X-radiation. On the indicated days after irradiation, cells were processed for ChIP analysis using antibodies to SIRT1. The levels of the promoter regions of IL-8 (left) and IL-6 (right) were determined by quantitative RT-PCR analysis and shown as fold enrichment by 0h. Data are means ± s.d. of triplicates from experiments that were repeated at least twice with similar results. ∗, p<0.05 Student’s *t*-test. **B and C**, Cells were treated as in A and were processed for ChIP analysis using antibodies to H3-K9ac (left) or H4-K16ac (right). The levels of the promoter regions of IL-8 (B) and IL-6 (C) were determined by quantitative RT-PCR analysis and shown as fold enrichment by 0h. Data are means ± s.d. of triplicates from experiments that were repeated at least twice with similar results. ∗, p<0.05 Student’s *t*-test.

We examined whether the ectopic expression of SIRT1 markedly repressed the promoter regions of SASP components. The amount of SIRT1 binding to the promoter regions of IL-8 and IL-6 genes was markedly higher in SIRT1-expressing cells than in control cells (Fig. 7A). In spite of the enriched binding of SIRT1, the acetylation levels of histone H3 (K9) and H4 (K16) on the promoter regions of IL-8 and IL-6 genes in SIRT1-expressing cells were similar to those in control cells (Fig. 7B). Upon the induction of senescence, SIRT1 binding to these regions in SIRT1-expressing cells was markedly decreased and became almost equal to that in senescent control cell (Fig. 7A). Although the acetylation levels of histone H3 (K9) and H4 (K16) on these regions in SIRT1-expressing cells also increased, no significant differences were observed from those in control cells (Fig. 7B). These results demonstrated that ectopic SIRT1 did not modify histone acetylation levels on the promoter regions of SASP components because SIRT1 dissociated from those regions during the induction of senescence.

## Discussion

SASP exhibits some beneficial functions including tumor suppression, the regulation of tissue repair, such as wound healing, and fibrosis, but also induces tumor progression, inflammation, and tissue degeneration, which promotes cancer, several age-related diseases, and organism aging [40–42]. In the present study, we showed that SIRT1, a well-known regulator of age-related diseases, regulated the expression of SASP components by silencing their promoter regions through epigenetic gene regulation. Although SIRT1 has been shown to repress repetitive DNA and several sets of genes that cross the whole genome by epigenetic regulation, SIRT1 dissociated the chromatin structure, thereby derepressing SIRT1-mediated genes during aging and in response to DNA damage [32]. We herein demonstrated that SIRT1 bound to the promoter regions of IL-8 and IL-6, major components of SASP, while SIRT1 dissociated from these regions in response to the induction of senescence. The acetylation of Histone H3 (K9) and H4 (K16) in the IL-8 and IL-6 promoter regions gradually increased during cellular senescence (Fig. 6A). The acetylation levels of these regions were already higher in SIRT1-depleted cells than in control cells in the pre-senescent stage. These acetylation levels were also significantly higher in SIRT1-depleted cells than in control cells during cellular senescence (Fig. 6B and C). In SIRT1-overexpressing cells, the binding of SIRT1 to the promoter regions was markedly increased in the pre-senescent stage, while the acetylation levels of promoter regions were similar to those in control cells (Fig. 7A). In response to the induction of senescence, SIRT1 binding was decreased to almost the same level as that in control cells due to the DNA damage-mediated dissociation of SIRT1. The acelylation levels of promoter regions were also simiular to those in control cells (Fig. 7B and C). Therefore, the induction of IL-8 and IL-6 mRNAs was almost the same as that in control cells (Fig. 5). These results suggested that SIRT1 repressed the expression of SASP factors through the deacetylation of histones in their promoter regions. A previous study reported the epigenetic gene regulation of SASP components [19]. The DNA damage response provoked by the induction of senescence leads to the degradation of the methyltransferases, G9a and GLP, which reduces the levels of H3K9me2 around the IL-6 and IL-8 promoters, thereby inducing the expression of these factors. Taken together, these findings and the present results showed that DNA damage-mediated epigenetic gene regulation through the dissociation of histone deacetylase SIRT1 and degradation of the methyltransferases, G9a and GLP, play a pivotal role in the expression of SASP components during cellular senescence.

The expression of some SASP components such as IL-8 and IL-6 is known to be required for the sustained DDR-mediated activation of NF-κB [11–13,43,44]. A previous study reported that SIRT1 deacetylated and inactivated NF-κB [31]. Since SIRT1 protein levels were found to be markedly reduced during senescence (Figs. 1 and 2), SIRT1 may not be involved in the regulation of NF-κB activity under these conditions. The activity of SIRT1 is required for NAD^+^, an essential co-factor [45]. NAD^+^ concentrations were shown to be reduced by the activation of poly-ADP-ribose polymerases (PARPs), NAD^+^-consuming enzymes, in response to DNA damage. NAD^+^ levels were found to be systemically elevated in both PARP knockout mice and mice treated with PARP inhibitors [46]. Although SIRT1 protein levels were maintained during senescence in SIRT1 overexpressing cells, SIRT1 may not be active due to reductions in NAD^+^ concentrations during the induction of senescence. In support of this result, the induction of SASP components in SIRT1-overexpressing cells was similar to that in control cells (Fig. 5). Taken together, these results showed that SIRT1 activity was markedly decreased during the induction of senescence due to the degradation of SIRT1 and reductions in NAD^+^ levels; therefore, SIRT1 may not be involved in NF-κB activity in senescent cells.

DNA damage was recently reported to promote organism aging. DNA damage accumulated in stem cells from several tissues including hematopoietic stem cells and satellite cells, leading to their functional inability [47–52]. DNA damage also stimulated the DNA damage response and DNA repair machinery that activates the repair enzymes, PARPs, leading to a decline in NAD^+^ levels. Consistent with these findings, PARP activity was shown to be increased in aged worms and mice [53]. Thus, the chronic activation of PARPs induced a decline of NAD^+^ levels in elderly individuals. Previous studies reported that NAD^+^ levels were approximately two-fold lower in old worms and in multiple tissues, including the liver and skeletal muscle, in aged mice [53–55]. Thus, reductions in the level and activity of SIRT1 during aging and the DNA damage-initiated induction of senescence may promote increases in the levels of systemic inflammatory cytokines and chemokines, leading to the expansion of senescent cells. This may account for the low level chronic inflammation observed in elderly individuals, thereby increasing susceptibility to age-related diseases and promoting individual aging. Therefore, SIRT1 may protect against aging and age-related diseases by suppressing SASP through epigenetic gene regulation.
